# MRI Characteristics and Alterations in Patellar Height in Patients with Patellar Tendinopathy—A Retrospective Study

**DOI:** 10.3390/jpm13040698

**Published:** 2023-04-21

**Authors:** Kim Loose, Sophie Pennekamp, Wolfgang Hitzl, Maximilian Willauschus, Johannes Rüther, Sandeep Silawal, Philipp Schuster, Hermann Josef Bail, Michael Millrose, Markus Geßlein

**Affiliations:** 1 Department of Orthopedics and Traumatology, Paracelsus Medical University, Breslauer Straße 201, 90471 Nuremberg, Germany; sophie.pennekamp@stud.pmu.ac.at (S.P.); maximilian.willauschus@klinikum-nuernberg.de (M.W.); johannes.ruether@klinikum-nuernberg.de (J.R.); philipp_schuster@gmx.de (P.S.); hermann-josef.bail@klinikum-nuernberg.de (H.J.B.); m.millrose@icloud.com (M.M.); markus.gesslein@klinikum-nuernberg.de (M.G.); 2 Research and Innovation Management (RIM), Biostatistics and Publication of Clinical Trial Studies, Paracelsus Medical University, 5020 Salzburg, Austria; wolfgang.hitzl@pmu.ac.at; 3 Department of Ophthalmology and Optometry, Paracelsus Medical University Salzburg, 5020 Salzburg, Austria; 4 Research Program Experimental Ophthalmology and Glaucoma Research, Paracelsus Medical University, 5020 Salzburg, Austria; 5 Institute of Anatomy and Cell Biology, Paracelsus Medical University, Prof. Ernst Nathan Str. 1, 90419 Nuremberg, Germany; sandeep.silawal@pmu.ac.at; 6 Center for Sports Orthopedics and Special Joint Surgery, RKH Orthopedic Hospital Markgröningen, 71706 Markgröningen, Germany; 7 Department of Trauma Surgery and Sports Medicine, Garmisch-Partenkirchen Medical Centre, 82467 Garmisch-Partenkirchen, Germany; 8 GOTS (German-Austrian-Swiss Society for Orthopaedic Traumatologic Sports Medicine), Muscle and tendon Committee, 07743 Jena, Germany

**Keywords:** patellar tendinopathy, jumper’s knee, magnetic resonance imaging, patellar tendon thickness

## Abstract

(1) Background: Patellar tendinopathy (PT) is an overuse condition of the knee extensor mechanism characterized by ventral knee pain at the lower pole of the patella and limited functionality. (2) Methods: In this retrospective study, a group of patients with PT (*n* = *41*) was compared with a control group (*n* = *50*) in terms of patient-related data and magnetic resonance imaging (MRI) characteristics. (3) Results: Patellar height was higher in the PT patient group and there was a significant difference in Caton–Deschamps index (CD) compared to the control group (*p* = 0.021). Patients with PT showed a lower patella–patellar tendon angle (PPTA) (*p* = 0.011). The patellar tendon thickness (PTT) in the proximal (PTTprox), middle (PTTmid) and distal (PTTdistal) part of the tendon was significantly thickened (*p* < 0.001). Increased signal intensity in MRI was detected in symptomatic tendons over 6 months compared to a duration of less than 6 months (*p* = 0.025). A significant relationship between the PTTprox and an increased signal intensity was observed (*p* < 0.001). (4) Conclusions: Patients with PT showed a significant difference in the patellar height and PPTA. With persistence of symptoms over 6 months, MRI seems suitable to detect the morphologic tendon changes and further identify patients suitable for surgical procedures.

## 1. Introduction

Patellar tendinopathy (PT) is a common orthopedic overuse condition of the knee extensor mechanism characterized by anterior knee pain [1]. Repetitive eccentric quadriceps loading in sports such as volleyball, track and field, basketball, long distance running and skiing can lead to PT, also called jumper’s knee [1,2]. This overuse condition occurs more often in males and from adolescence through to the fourth decade of life. Up to 45% of elite jumping athletes will have PT at any point during their career [3,4]. A prospective study reported that 53% of athletes with PT were forced to quit competitive sport [5]. PT can also cause limitations in non-athletes [6].

Microtrauma of the tendon may lead to the degeneration of individual fibers due to tension in the tendon, which can accumulate over time and lead to chronic tendinopathy [7]. It is noticeable that the tendon does not recover from this degeneration/microtears. The exact pathogenesis of chronic tendinopathy remains to be definitively elucidated but is likely to develop independent of inflammation [8]. Histology shows intratendinous collagen degeneration, hypercellularity, neovascularization and, occasionally, calcifications and local necrosis [9]. PT is a clinical diagnosis; patients report progressive activity-related pain at the distal patellar pole [4]. Activities of daily living may be affected by climbing the stairs, squatting and prolonged sitting [4].

In the jumper’s knee classification (JKC) according to Blazina et al., PT develops in four stages: stage 1 is pain after activity without functional impairment, stage 2 is pain during and after activity but still with satisfactory performance, stage 3 is characterized by persistent pain during and after activity and difficult performance and stage 4 is tendon rupture [10]. Ultrasound and magnetic resonance imaging are suitable for diagnosing PT [1]. Conservative treatment is the initial treatment method for PT and includes pain modulation, stress management, stress progression and functional strengthening [6]. Surgical therapy is used if six months of extensive conservative management is unsuccessful [11]. Good results have been achieved with arthroscopic and open surgical procedures in refractory cases of PT [8]. Refractory response to treatment and long-term symptoms of PT are reported in several studies, making targeted prevention and adapted treatment of the sports injury all the more important [1,12].

In addition to extrinsic risk factors for PT, such as physical activity and training volume, there are known intrinsic risk factors such as patellar height, patellar thickness and increased patellar signal intensity [4]. Some of these patellofemoral instability measurements have been investigated in a few studies: in 1986, Kujala et al. compared athletes with patellar chondropathy (*n* = *20*), jumper’s knee (*n* = *20*) and a control group of symptom-free athletes (*n* = *20*) and showed an accumulation of patella alta associated with jumper’s knee (*p* < 0.05) [13]. This correlation was confirmed by Tscholl et al. in their case–control study [14]. Patellar height affects the pattern of the articulation of the patella with the femur during flexion and has an inconsistent effect on the biomechanics of the patellar tendon [15], which again can lead to tendinopathy.

In a study of elite university athletes with PT symptoms (*n* = *16*) compared to athletes with PT (*n* = *49*), Nishida et al. found a patellar tendon thickness of more than 7 mm on MRI to be significant [16].

In physiologic conditions, the patellar tendon appears on MRI as a homogeneous strip with low signal intensity and can be clearly distinguished from the infrapatellar fat pad. In patellar tendinopathy, there is a widening of the patellar tendon and a heterogenous increase in signal intensity [11].

Another discussed aspect in the etiology of PT is patellofemoral instability and trochlear dysplasia. Trochlear dysplasia results in a loss of bone stability of the patellofemoral joint [17], altering the distribution of force to the patellar tendon. In their study, Ivengar et al. found hypertrophy of the medial portion of the patellar tendon in patients with patellofemoral joint instability and trochlear dysplasia. Fifty MRIs of the knee joint with trochlear dysplasia and fifty MRIs with normal patellofemoral joint morphology were compared [18].

The understanding of PT by analyzing different measurements on MRI is crucial for the improvement of treatment. Regarding the MRI measurements, there has so far been limited data with concrete thresholds and any large potential.

The primary aim of the study was to evaluate if typical patellofemoral instability measurements on MRI correlate with the presence of PT to better guide the prevention and treatment of a protracted injury. The study further investigated whether there is a correlation of PT with symptoms over 6 months and the radiological image.

## 2. Materials and Methods

### 2.1. Study Population

**Figure 1:**
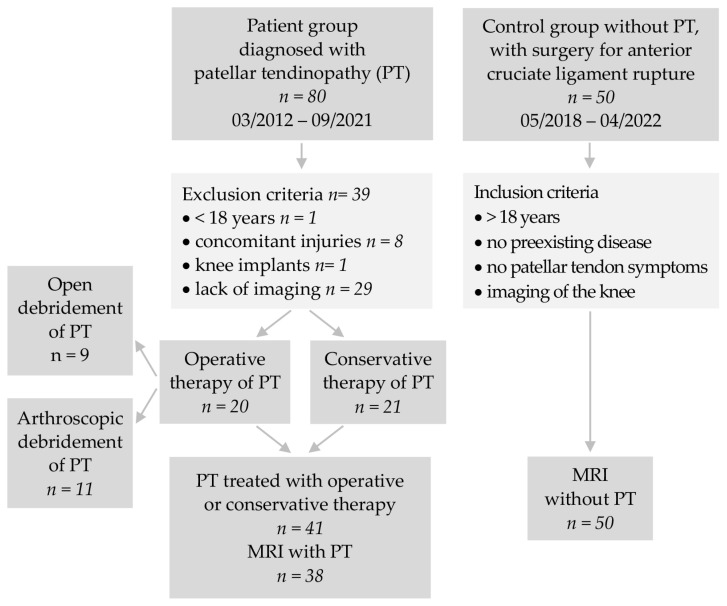
Study flowchart.

From a total of 80 patients with PT treated in the period from March 2012 to September 2021 at a maximum care hospital, 41 patients were included in the study for a retrospective analysis (Figure 1). The control group consisted of 50 patients who underwent surgery for anterior cruciate ligament rupture without any prior symptoms of anterior knee pain at the same maximum care hospital from May 2018 to April 2022. The control group was chosen as all cases had standard MRIs and usually represent a comparable age group.

Inclusion criteria were a minimum patient age of 18 years and surgical (arthroscopic debridement or open debridement) or conservative treatment of PT. The patients considered in the study were affected only unilaterally and not bilaterally. Exclusion criteria were concomitant injuries that affected the ipsilateral knee, patients with existing knee implants or prior surgery on the knee, as well as lack of imaging. The control group included patients 18 years of age and older, without preexisting disease or patellar tendon symptoms, and with imaging of the knee.

### 2.2. Diagnosis and Treatment

Patient history, clinical examination with ultrasound, X-rays- and/or MRI -examination followed by an adequate diagnosis of all patients were undertaken by specialists at the orthopedic trauma surgery outpatient clinic. PT was treated either conservatively or surgically if symptoms persisted for more than six months. Conservative therapy was the treatment of choice in early stages and at the point of initial diagnosis. It consisted of physiotherapy with manual therapy and, in sports therapy, eccentric training of the quadriceps femoris muscle. In cases of persisting symptoms after conservative therapy, surgery was performed by an experienced orthopedic surgeon. The applied surgical techniques were either arthroscopic debridement or open debridement of the patellar tendon. Postoperatively, the affected leg was put in an orthosis with the leg fully extended. All patients were mobilized with partial weight bearing using crutches on the second day after surgery under instructions from a competent physiotherapist. Full loading of the leg was allowed after 7 days. Patients were instructed to wear the knee straightening brace with a ROM 0-0-90 for 2 weeks. Physiotherapy was allowed after one week. Full knee flexion and active knee extension were allowed after 6 weeks.

### 2.3. Patient-Related Data

Patient-related data such as gender assigned at birth, age at diagnosis in years, body mass index (BMI), sports, stage of the disease classified according to the JKC and duration of complaints under or over 6 months were extracted from hospital medical records.

### 2.4. Evaluation of Preoperative MRI

Preoperative MRI was available for evaluation in 38 patients with PT and in 50 patients in the control group. Images were acquired using 1.5 or 3.0 Tesla scanners. T1-, T2- and proton-weighted images were considered. The sequences used were SE (spin-echo), SE fs (spin-echo fat saturation), TSE (turbo spin-echo) and STIR (short-tau inversion recovery). Sagittal and axial cross-sectional images were evaluated. Images were evaluated retrospectively on an approved PACS workstation (Ashvins, MedicalCommunications, Heidelberg, Germany).

### 2.5. Measurement Methods in MRI

**Figure 2:**
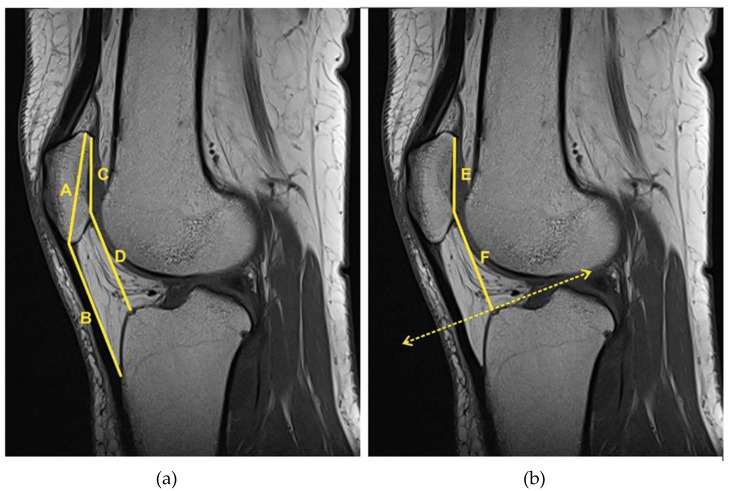
(**a**) Insall–Salvati index (IS) (B/A): patellar tendon length from the lower pole of the patella to the tibial tuberosity (B) to the diagonal length of the patella (A). Caton–Deschamps Index (CD) (D/C): distance from the inferior edge of the articular surface of the patella to the anterior edge of the tibial plateau (D) to the articular surface of the patella (C). (**b**) Blackburne–Peel index (BP) (F/E): distance between the tibial plateau line and the inferior point of the patellar articular surface (F) to the length of the patellar articular surface (E).

The Insall–Salvati index (IS) (B/A), Caton–Deschamps index (CD) (D/C) and the Blackburne–Peel index (BP) (F/E) were applied to determine patellar height (Figure 2).

**Figure 3:**
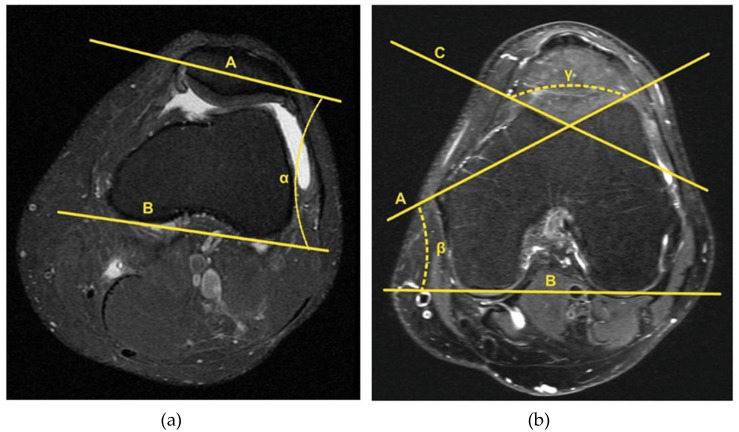
(**a**) Patella tilt angle (PTA) = α: angle between a straight line through the transverse axis of the patella (A) and a tangent along the posterior femoral condyles (B). (**b**) Lateral trochlear inclination angle (LTI) = β: angle between the tangent along the facies patellaris femoris lateralis (A) and the posterior condylar tangential line (B). Sulcus angle (SA) = γ: angle between the lateral facies patellaris femoris (A) and the medial facies patellaris femoris (C).

In the axial plane, the patella tilt angle (PTA) was measured in the sectional view with the greatest spread of the patella from medial to lateral using the Dejour method (Figure 3a). To describe the trochlea and possible trochlear dysplasia, the lateral trochlear inclination angle (LTI) and the sulcus angle (SA) were obtained in the axial plane. Both measurements were performed in the image with the greatest distance between the intercondylar fossa and the facies patellaris femoris. The LTI describes the angle between the facies patellaris femoris lateralis and posterior condylar tangential line. The SA is formed from the angle between the lateral and medial facies patellaris femoris (Figure 3b).

**Figure 4:**
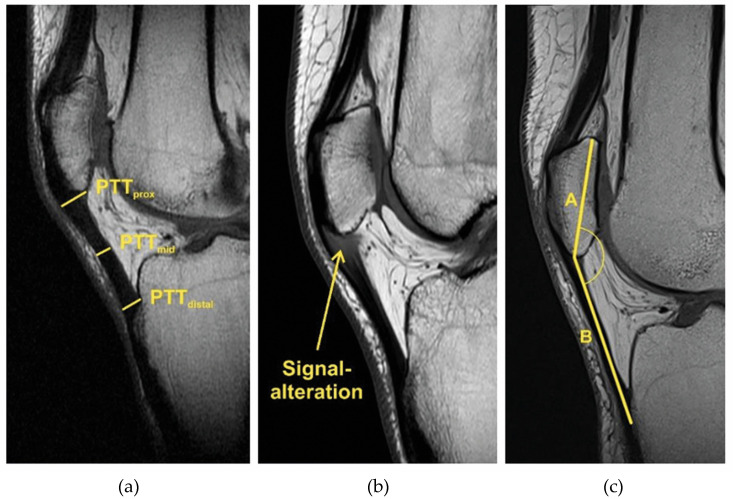
(**a**) Patellar tendon thickness (PTT): shown here on MRI, a thickened patellar tendon with PT in the proximal (PTTprox), middle (PTTmid) and distal segments (PTTdistal). (**b**) Signal intensity (alteration) in PT at the origin of the patellar tendon. (**c**) Patella–patellar tendon angle (PPTA): angle between the line drawn from the base of the patella to the apex (A) and the line drawn between the apex of the patella to the insertion of the patellar tendon on the tibial tuberosity (B).

Patellar tendon thickness (PTT) was measured in the sagittal plane on the MRI image with the greatest distance between the base patella and apex patella. Tendon thickness was measured proximally at the origin of the tendon (PTTprox), distally at the insertion at the tibial tuberosity (PTTdistal) and at the midpoint of these two points (PTTmid) (Figure 4a). In the same image, the presence of signal intensity was also checked (Figure 4b). The patella–patellar tendon angle (PPTA) measures the angle between the line drawn from the base of the patella to the apex (A) and the line drawn between the apex of the patella to the insertion of the patellar tendon on the tibial tuberosity (B) (Figure 4c).

### 2.6. Statistical Analysis

All data were obtained and analyzed retrospectively and the statistical analysis was conducted with IBM SPSS Statistics version 28.0 (version 28, Armonk, NY, USA). For metric parameters, Levene’s test of equality of variance and bootstrap test with 4000 samples and 95% confidence interval were applied. For nominal variables, Pearson’s chi-square test or Fisher’s exact test were used. In addition, the least significant difference test (LSD test) was applied to represent significant differences. The *p*-values are two-sided and considered statistically significant at an alpha level of <0.05. Results are presented as mean ± standard deviation and range with associated minimum and maximum.

The study was approved by the institutional review committee (Paracelsus Medical University at the Nuremberg Hospital, Number IRB-2022-007) following national legal guidelines.

## 3. Results

A total of 91 patients were considered, of which 41 had PT (patient group) and 50 were without PT (control group).

### 3.1. Demographic Data

On average, patients with PT were 31.5 years old and had a BMI of 25.6 (Table 1). A total of 31.7% of the patient group was female and 68.3% was male. There was no significant difference comparing the genders of both groups (*p* = 0.976). PT was localized on the left side in 43.9% and on the right side in 56.1%, thus the probability of PT was equal on both sides (*p* = 0.295). There was equality of means in all listed patient-related data and no significant difference between patient and control group (*p* > 0.05). Thus, the two groups were well comparable (Table 1).

A total of 78.0% of patients with PT (32 of 41) participated in popular or professional sports, and 22.0% (9 of 41) did not participate in any sports. Soccer (22.0%) was the most popular sport. Among others, a variety of ball sports were present (39.1%) as well as running (12.2%) and martial arts (9.7%).

There was a significant correlation (Pearson’s chi-squared test, *p* < 0.001) between the stage of the disease, classified according to the JKC, and the duration of symptoms under or over 6 months. The higher the stage of PT, the longer the symptoms persisted in patients. Thirty-three of the forty-one patients with PT showed symptoms for more than 6 months, and eight patients less than 6 months. All patients suffered from functional impairment.

### 3.2. Biomechanical Differences between Patient and Control Group in MRI

#### 3.2.1. Patellar Height

For the indices of patellar height, the MRI evaluation showed a significant difference in the Caton–Deschamps (CD) between the patients and control group, a borderline significant finding in the Blackburne–Peel (BP), and no significant difference in the Insall–Salvati (IS) indices (Table 2).

#### 3.2.2. Patella–Patellar Tendon Angle (PPTA) and Patellar-Tilt Angle (PTA)

PPTA was found to be significantly lower in the patient group with PT at 142.8° ± 4.8° (vs. 145.7° ± 5.5°) (*p* = 0.011). No significant difference was found in PTA between the patient group (6.6° ± 4.3°) and the control group (7.8° ± 4.9°) (*p* = 0.214).

#### 3.2.3. Trochlear Dysplasia

There were no significant differences in trochlear dysplasia measured in sulcus angle (SA) and lateral trochlear inclination angle (LTI) between the patient group and control group (*p* > 0.05).

#### 3.2.4. Patellar Tendon Thickness (PTT) and Signal Intensity

The patellar tendon was widened in all tendon segments in the patient group and was significantly thicker than in the control group (*p* < 0.001) (Table 3). In the proximal portion (PTTprox), the mean value in PT was 6.83 mm, almost 2 mm higher than that of the control group. It was the largest mean value for PTTprox in the patient group compared to the other tendon sections. The mean tendon thickness was 5.34 mm, and the smallest mean value measured in PT as well as the same pattern seen in the control group was 3.73 mm. PTTdistal was the largest measured mean value in the control group with 4.98 mm.

The increased signal intensity occurred in 78.9% of the cases with PT (30 of the 38 patients). This was significantly higher (*p* < 0.001) than the control group (10%, 5 of the 50 patients). Patients with surgical therapy of PT had significantly more increased signal intensity of PT compared with patients without surgical therapy (*p* = 0.017) (Table 4).

### 3.3. Correlation of Stage of Disease and Radiological Image

Increased signal intensity in chronic PT (>6 months) occurred in 87.1% of patients (*n = 27*) compared with 42.86% of patients with PT lasting less than 6 months (*n = 3*) (*p* = 0.025). In PT of less than 6 months, no increased signal intensity was present in 57.14% (*n = 4*). The patellar height correlated with higher PTTprox, however, not significantly. There was a significant correlation between PTTprox and the presence of increased signal intensity (*p* < 0.001). In the patients with PT, increased signal intensity was present in 30 of 38 cases with a PTTprox of 7.30 ± 2.39 mm. The 8 of 38 cases without increased signal intensity showed a significantly lower PTTprox of 5.09 ± 1.20 mm.

## 4. Discussion

The main findings of this study were that the patellar height was significantly higher, the patella–patellar tendon angle (PPTA) was significantly lower and the patellar tendon thickness (PTT) was significantly thicker in the patients group compared to the control group. Furthermore, the risk of an increased signal intensity of patellar tendon at symptoms > 6 months was higher than with symptoms < 6 months.

Patella alta is considered a risk factor of PT in the literature [19]. In this study, the indices of patellar height in MRI evaluation showed a significant difference in the Caton–Deschamps (CD) between the patients and control group and a borderline significant finding in the Blackburne–Peel (BP). Thus, this study is in agreement with the study by Tscholl et al., in which patients with PT had higher patellar scores in CD (1.17 ± 0.17) [14]. In contrast to other measured indices, the IS index (Table 2) does not show a high significant difference between the patient and control groups. This could be explained by the fact that the original measure was evaluated on plain lateral X-ray images and there might be a certain loss of resolution for the exact measurement in the MRI T1 imaging [20]. This study confirms the hypothesis of a higher patella as an intrinsic risk factor for PT. Thus, especially in athletes from sports such as volleyball, soccer, or basketball and either asymptomatic or in the early stages of anterior knee pain, the measurement of patellar height may prove useful to prevent the risk of chronic PT and the potential functional consequences, up to and including withdrawal from sport. Patella alta is caused in part to a shortening of the quadriceps femoris muscle due to muscle tension, which underscores the importance of eccentric training and physiotherapy as therapeutic options for PT.

In this study, PPTA was found to be significantly lower in the patient group with PT than in the control group. Comparable studies to PPTA in PT were not found in the literature research. Published work considers PPTA in the context of other patellofemoral conditions such as medial patellar plica syndrome, chondromalacia patellae and infrapatellar fat pad syndrome. The angle was found to be lower in patients with anterior knee pain caused by one of these above stated diagnoses than in the control groups [21]. A biomechanical reason for the lower PPTA could be that the infrapatellar fat pad undergoes a fibrosis during nonphysiological loading [21].

In this study, all tendon segments of the PTT had significant thickening in comparison to the control group. A widened patellar tendon, especially in the proximal portion of the tendon, is considered a typical characteristic of PT [16,22]. Various thresholds for patellar tendon thickness PTT have been described in the literature [16,22,23]. El-Khoury et al. found that the mean patellar tendon thickness of the healthy participants (*n = 10*) was 3.7 mm, whereas that of the patients (*n = 50*) to have been 10.9 mm [23]. In this study, the mean value of the control group for PTTprox was 4.76 ± 0.97 (2.84–7.02). The results of Nishida et al. showed that PTT thicker than 7.0 mm has clinical significance on both US and MRI [16]. In this study, patients with PT had a PTTprox of 6.83 ± 2.36 (3.67–13.59). Nishida et al. described in their work the PTTmid as 4.6 ± 0.9 mm (*p* = 0.005), while in this study, it was 5.34 ± 1.54 mm, which coincided with the observation that in this tendon section, there was also a significantly larger value for the PT. The trend of a significantly thickened tendon was also seen in the PTTdistal in this study, but this contradicts the observations of Nishida et al. who did not show any significant differences in the distal portion (*p* = 0.67) [16]. It would be beneficial to measure the patellar tendon thickness divided into PTTprox, PTTmid and PTTdistal by MRI in further studies to generate clear cut-off values.

In their review, Dan et al. examined the relationship between biomechanics of the knee extensor mechanism and its relationship to PT. They claim that so far, no intrinsic morphological risk factors of the patella for patellar tendinopathy can be identified. Biomechanically, the patella should be considered as a lever in the sagittal plane and this should be considered as a basis for identifying morphological intrinsic risk factors [15]. In a subsequent study by Dan et al., based on measurements from lateral radiographs, the patellar tendon in patients with patellar tendinopathy (*n = 52*) had smaller lever and moment arms than in the control group (*n = 53*). What is striking in this study is that in the various measurements of patellofemoral instability mentioned above, only the sagittal measurements yielded significant results. The measurements in the axial planes remained without significance. The previously mentioned information supports the thesis of Dan et al. in that the patella should be seen as a lever in the sagittal plane and is also related to the transmission of force to the tibia.

In this study, the risk of an increased signal intensity of PT at symptoms > 6 months was higher than with symptoms < 6 months. It has been described in the literature that MRI shows an increased T2 signal intensity within the patellar tendon in degenerative changes [11]. An increased signal intensity in T1 and T2 at the patellar tendon origin at the lower pole of the patella in this study was observed in 30 of the 38 cases from the patient group. Additionally, there was a significant correlation between PTTprox and the presence of increased signal intensity. The thicker the patellar tendon, the more often there is increased signal intensity, indicating that there are probably also different stages of PT. These could, in addition to the JKC according to Blazina et al., represent a way of classifying the overuse condition, which requires further research. In contrast to JKC, this would be a more objective and reproducible method to assess the severity of PT and to plan a further therapeutic approach.

The results of this study indicate that if conservative treatment fails, a radiological examination by MRI should be performed at the latest after 6 months of persistent symptoms. Furthermore, surgical therapy can be considered at this stage since morphological changes in the tendon are already present by then.

Surgical procedures for PT can generally be performed open or arthroscopically based on the surgeon’s preference. Tendon necrosis is mostly identified on MRI centrally within the tendon in the proximal to mid-substance parts, requiring careful surgical debridement. In those cases, the authors favor an open longitudinal surgical approach to ensure complete excision of the affected tissue and secure tendon sheet closure with resorbable sutures. During surgery, special attention should be paid to the resection of a bony “patellar nose” which can occur on the distal patellar pole and which often causes bony edema and impingement to the proximal patellar tendon and is visible on MRI as a bone bruise on the patellar side with visible adjacent inflammatory tendon changes on the T2 weighted images.

As the study highlights the role of patella alta, the possibility of patella lowering tuberosity osteotomy in cases of chronic PT should be further discussed. Besides lowering the patella height, additional elevation of the tuberosity could also increase the PPTA and improve the surgical outcome. However, as the exact role of an elevated PPTA in this context remains unclear, further studies on the clinical implications are necessary.

Therefore, physiotherapy remains the main focus and should aim on altering individual risk factors such as patellar height. Targeted training of the posterior thigh and gluteal muscles, coordination training for the thigh extensor and stretching of the anterior thigh muscles are viable options [24].

### Limitations

The study had several limitations. It was a retrospective study and the results were dependent on the quality and quantity of patient data collected. It was a single-center study, which calls into question the generalizability of the results. The case number of 41 patients with PT was small; for example, some patients had to be excluded from the collective due to lack of imaging. Many studies on PT investigated professional athletes with high jump loads, whereas in this study, a wide range of different sports and mainly amateur athletes were represented. In the surgically treated patients, it must be noted that there are often several weeks to months between the radiological examination of the knee and the operation, which means that the extent of the disease may not have been adequately represented in the MRI.

Compared to other studies, the control group is an aspect worth emphasizing, because with an equality of means in demographic data, the two groups were comparable. Various measurements were performed on the patellar tendon, patella, trochlea and patellofemoral joint. This broad view can be cited as a strength of the study, as can the fact that conservatively and surgically treated patients were considered, thus representing a wide range of injury stages.

## 5. Conclusions

Patients with PT showed a significant increase in the patellar height, tendon thickness and PPTA. Conservative therapy should focus on the early stages of improvement of those individual risk factors. With a persistence of symptoms of over 6 months, MRI seems suitable to detect the further morphologic tendon changes as a thickening or partial necrosis and further identify patients suitable for surgical procedures.

## Figures and Tables

**Table 1:** Patient-related data.

	Patients with PT	Control Group	*p*-Value
Number of patients (*n*)	41	50	>0.05
Gender, female (*n*)	13	16	0.976
Gender, male (*n*)	28	34	0.976
Age, y (mean ± SD)	31.5 ± 11.9	30.9 ± 8.1	0.784
BMI ^1^ (mean ± SD)	25.6 ± 5.6	25.9 ± 4.8	0.826

^1^ BMI: body mass index.

**Table 2:** Comparison of patellar height on MRI.

	Patients with PT *n = 38*	Patients without PT *n = 50*	*p*-Value
IS ^1^ (mean ± SD (range))	1.21 ± 0.19 (0.78–1.57)	1.17 ± 0.14 (0.93–1.57)	0.244
CD ^2^ (mean ± SD (range))	1.22 ± 0.19 (0.82–1.77)	1.13 ± 0.15 (0.86–1.51)	0.021
BP ^3^ (mean ± SD (range))	1.14 ± 0.18 (0.77–1.46)	1.07 ± 0.16 (0.75–1.47)	0.053

^1^ IS = Insall–Salvati index; ^2^ CD = Caton–Deschamps index; ^3^ BP = Blackburne–Peel index.

**Table 3:** Comparison of patellar tendon thickness (PTT) and signal intensity in the MRI of patient versus control group.

	Patients with PT *n = 38*	Patients without PT *n = 50*	*p*-Value
PTTprox ^1^, mm (mean ± SD (range))	6.83 ± 2.36 (3.67–13.59)	4.76 ± 0.97 (2.84–7.02)	<0.001 *
PTTmid ^2^, mm (mean ± SD (range))	5.34 ± 1.54 (3.30–9.80)	3.73 ± 0.66 (2.22–5.25)	<0.001 *
PTTdistal ^3^, mm (mean ± SD (range))	6.15 ± 1.61 (3.64–9.26)	4.98 ± 1.07 (3.29–7.30)	<0.001 *
Signal intensity, yes/no	30/8	5/45	<0.001 *

^1^ PTTprox = proximal patellar tendon thickness; ^2^ PTTmid = middle patellar tendon thickness; ^3^ PTTdistal = distal patellar tendon thickness; * significant.

**Table 4:** Comparison of patellar tendon thickness (PTT) and signal intensity in the MRI of patients with and without surgical therapy of PT.

	Patients with Surgical Therapy of PT *n = 19*	Patients without Surgical Therapy of PT *n = 19*	*p*-Value
PTTprox ^1^, mm (mean ± SD (range))	7.61 ± 2.89 (3.81–13.59)	6.05 ± 1.37 (3.67–9.13)	0.118
PTTmid ^2^, mm (mean ± SD (range))	5.80 ± 1.76 (3.35–9.80)	4.89 ± 1.16 (3.30–8.57)	0.129
PTTdistal ^3^, mm (mean ± SD (range))	6.14 ± 1.76 (3.64–9.00)	6.16 ± 1.50 (3.73–9.26)	0.861
Signal intensity, yes/no	18/1	12/7	0.017 *

^1^ PTTprox = proximal patellar tendon thickness; ^2^ PTTmid = middle patellar tendon thickness; ^3^ PTTdistal = distal patellar tendon thickness; * significant.

## Data Availability

Not applicable.
